# Rumen-Protected Leucine Improved Growth Performance of Fattening Sheep by Changing Rumen Fermentation Patterns

**DOI:** 10.3390/microorganisms13102377

**Published:** 2025-10-15

**Authors:** Shu Li, Jiantao Zhao, Peng Zhang, Shaoyang Pang, Mingyu Ma, Yifan Nie, Zhenzi Xu, Sijin Li, Yuanyuan Li, Wenju Zhang

**Affiliations:** College of Animal Science and Technology, Shihezi University, Shihezi 832000, China; lishu00520@163.com (S.L.); zz1513192544@163.com (J.Z.); 15297594947@163.com (P.Z.); 15251239380@163.com (S.P.); 18638694160@163.com (M.M.); nieyifan2001@outlook.com (Y.N.); 19882955630@163.com (Z.X.); lisijin0873@163.com (S.L.); liyuanyuan234@shzu.edu.cn (Y.L.)

**Keywords:** branched-chain amino acid, rumen, fermentation parameters, free amino acids, microbiota

## Abstract

This experiment investigated the effects of rumen-protected leucine (RP-Leu) supplementation on growth performance, rumen fermentation parameters, and rumen microbiota of fattening sheep. Forty fattening sheep (19.03 ± 0.38 kg) were randomly divided into four groups. The four groups were fed the basal diet supplemented with RP-Leu at 0 (L-0), 0.5 (L-0.5), 1.0 (L-1.0), or 1.5 (L-1.5) g/d. The experimental period lasted 67 d. The results demonstrated that compared with the L-0 group, the L-1.0 and L-1.5 groups significantly increased the average daily gain (ADG) by 22.2% and 18.0%, respectively (*p* < 0.05), and reduced the feed-to-gain ratio (F:G ratio) by 20.0% and 13.4% (*p* < 0.05); the L-1.5 group significantly increased the concentrations of propionate and isovalerate (*p* < 0.05) and significantly decreased the acetate to propionate ratio (A:P) by 25.7% (*p* < 0.05); and the L-1.0 group significantly decreased the concentrations of free branched-chain amino acids (BCAAs) in rumen (*p* < 0.05). A microbiota analysis showed that RP-Leu enriched the abundance of fiber-degrading bacteria. A correlation analysis showed that acetic acid and A:P were positively correlated with *norank_f_F082* and *norank_f_UCG-011* (*p* < 0.05). Phenylalanine, isoleucine, valine, and leucine were negatively correlated with *norank_o_Bacteroidales* (*p* < 0.05). In conclusion, RP-Leu improved the growth performance of fattening sheep by changing the rumen fermentation parameters and patterns; the optimal supplementation level is 1.0 g/d.

## 1. Introduction

The rumen is crucial for ruminant metabolism, production, and health [1], as it enables efficient extraction of nutrients from the diet through microbial fermentation [2]. However, the extensive ruminal degradation of feed-derived crude protein and amino acids often limits their availability for absorption, necessitating the use of rumen-protected amino acids (RPAAs) in fattening sheep production [3,4]. Among the RPAAs, branched-chain amino acids (BCAAs) have received particular attention. The current research on BCAAs primarily focuses on ruminants such as goats and dairy cattle, yet no consensus exists regarding their effects on modulating ruminal fermentation parameters and patterns [5].

Leucine (Leu), identified as the predominant branched-chain amino acid (BCAA) within animal systems [6,7], regulates mammalian protein synthesis, skeletal muscle protein turnover, and intestinal development [8,9]. Being an indispensable functional amino acid for ruminants, Leu is principally derived from three sources: rumen microbial crude protein (MCP), rumen undegraded dietary protein, and dietary supplements. However, its research progress in rumen function is limited [10]. Evidence for the importance of BCAAs in rumen function nevertheless comes from both in vitro and in vivo studies. In vitro rumen fermentation studies demonstrated that BCAA removal inhibits ruminal bacterial growth, reduces neutral detergent fiber (NDF) degradation and MCP synthesis, and impairs overall fermentation [11,12]. Conversely, in vivo trials with Hu sheep revealed that BCAA supplementation increased ruminal papillae length and total volatile fatty acid (TVFA) concentration while decreasing ammonia nitrogen (NH_3_-N) levels [13]. These findings indicated that BCAA had effects on rumen microorganisms and fermentation parameters. Specifically, for Leu, its mechanism of impact may involve its degradation product, isovaleric acid, which serves as a critical growth factor for many rumen fibrolytic microorganisms [12,14], enhancing ruminal fermentation rates, increasing volatile fatty acid (VFA) concentrations, and altering fermentation patterns. Supporting this, additional studies have shown that rumen-protected leucine (RP-Leu) elevated *Ruminiclostridium*, *Ruminococcaceae*, and *Pseudobutyrivibrio* abundance in rumen [14]. Furthermore, accumulating evidence indicates that BCAA supplementation improves the degradation of forage dry matter (DM), acid detergent fiber (ADF), and NDF [14,15]. It is important to note that the optimal concentration of BCAA is critical for ruminal microbial growth [16], as deficient or excessive levels of BCAAs or individual BCAA can adversely affect rumen microbes [5,17]. Despite these advances, the current research on RP-Leu mainly focuses on intestinal development and milk composition in sheep, with limited investigations into its effects on rumen fermentation function in fattening sheep.

Therefore, this experiment aimed to supplement different levels of RP-Leu in fattening sheep diets to (1) systematically evaluate the effect of RP-Leu on the growth performance of fattening sheep, (2) elucidate its regulatory effect on rumen fermentation parameters, and (3) analyze the potential mechanism of RP-Leu in remodeling the rumen microbial community. These objectives provide a theoretical basis and technical parameters for precision amino acid nutrition strategies in fattening sheep production systems.

## 2. Materials and Methods

### 2.1. Animal Ethics

Approval for the experimental procedures was granted by Shihezi University’s Animal Ethics Committee following comprehensive review.

### 2.2. Experimental Animals and Group Design

The experimental plant site was located in Gongnais Breeding Sheep Farm, Ili Kazakh Autonomous Prefecture, Xinjiang, China. Forty healthy four-month-old Xinjiang fine-wool sheep with similar weight (19.03 ± 0.38 kg) underwent random allocation into four experimental treatments. Each treatment contained ten sheep, with each sheep fed individually. The initial daily feed offer was set at 4% of the average body weight of each sheep and was adjusted daily thereafter to ensure ad libitum consumption while maintaining approximately 10% orts. The control group (L-0) received the basal diet, while the remaining three groups were added with different levels of RP-Leu to the basal diet: 0.5 g/d (L-0.5), 1.0 g/d (L-1.0), and 1.5 g/d (L-1.5). The supplementation level of RP-Leu was referenced from previous studies [18,19]. The daily amount of RP-Leu for each sheep was thoroughly mixed into the total daily ration and divided equally into two meals. Diets were administered twice daily (08:00 and 18:00), and all fattening sheep had free access to water. An adaptation period of 7 days was followed by a 60-day experimental period. The basal diet of each group was formulated according to the Nutritional Requirements of Sheep for Meat in China (NY/T 816-2021) [20] to meet all normal growth and nutrition requirements for fattening sheep. Feed ingredients and nutritional composition were detailed in Table 1.

Leu (purity ≥ 99.4%) was purchased from Hebei Huayang Biotechnology Co., Ltd. (Hengshui, China) Crystalline Leu was microencapsulated by Hangzhou Kangdequan Feed Co., Ltd. (Hangzhou, China) with a Leu content of 51.8%. The retention rate of simulated rumen fluid was 92.7% for 12 h, and the release rate of simulated intestinal fluid was 88.6% for 12 h.

### 2.3. Sample Collection

At the end of the trial, a random subset of four sheep per treatment group underwent euthanasia for slaughter after 12 h fast and 2 h water withdrawal. Pre-slaughter, the live weights were recorded. During evisceration, rumen fluid samples were collected, and four layers of sterile gauze were used to filter them. The resulting filtrate pH values were quantified employing a pre-calibrated pH meter (PHSJ-3F, Yidian Scientific Instruments Co., Ltd., Shanghai, China). The appropriate amount of rumen fluid was dispensed into freezer tubes and stored at −80 °C for the subsequent determination of rumen VFA and microbial analyses.

### 2.4. Growth Performance

The live weight of each sheep was recorded at the beginning and end of the formal experimental period, designated as initial body weight (IBW) and final body weight (FBW), respectively. These metrics were used for the computation of average daily weight gain (ADG). Individual daily feed consumption and refusals were monitored and recorded for each sheep throughout the trial to calculate the average dry matter intake (ADMI).

### 2.5. Apparent Nutrient Digestibility

The apparent digestibility was determined according to Van Keulen, J. et al. (1977) [21], with some modifications. Fecal samples (300 g per sample) were acquired daily for three days prior to trial termination, four hours post-morning feeding. Corresponding feed samples (300 g) were concurrently obtained. Fecal samples (10–20 g) were weighed into crucibles and ashed at 650 °C for 5 h. The resulting ash was moistened with 5 mL of deionized water, treated with 10 mL of concentrated HCl, and evaporated to dryness in a water bath. This acid treatment was repeated with 5 mL of concentrated HCl, followed by 5 min incubation in a water bath and filtration. Acid residues were removed by rinsing with hot distilled water. The residue and filter paper were transferred to a crucible, re-ashed at 650 °C overnight, and then the crucible was cooled and weighed to determine the AIA content.

### 2.6. Rumen Fermentation Parameters

Concentrations of ruminal volatile fatty acid (VFA) were quantified according to Lu et al. (2025) [22]. Thawed rumen fluid was centrifuged (3000× *g*, 4 °C, 10 min), and 1 mL of supernatant was transferred to a 2 mL centrifuge tube wherein 200 µL 25% metaphosphoric acid was added. Subsequently, the mixture was centrifuged (10,000× *g*, 4 °C, 10 min) and filtered through a membrane into a feed bottle. Quantification of ruminal volatile fatty acids (VFAs) was performed using a gas chromatograph (7890B, Agilent, Santa Clara, CA, USA). NH_3_-N concentration was determined according to Peng et al. (2024) [23]. Rumen fluid (10 mL) was centrifuged (3500× *g*, 10 min), and 2 mL of the supernatant was mixed with 8 mL of 0.2 mol/L hydrochloric acid to obtain a 5-fold dilution. Then, 0.4 mL of the diluted sample was transferred to a clean test tube, followed by the sequential addition of 2 mL of reagent A (0.08 g sodium nitroprusside dissolved in 100 mL of 14% sodium salicylate solution) and 2 mL of reagent B (2 mL sodium hypochlorite solution mixed with 100 mL of 0.3 mol/L sodium hydroxide solution). The mixture was thoroughly vortexed and allowed to stand for 10 min for color development. Absorbance was measured at 700 nm using a spectrophotometer. A standard curve was constructed using ammonia nitrogen standard solutions in the concentration range of 0–1.2 mg/100 mL treated under the same conditions, and a regression equation was derived. The ammonia nitrogen content in the sample was calculated by substituting the measured absorbance into the regression equation and multiplying by the dilution factor.

### 2.7. Ruminal Free Amino Acids

According to the method of Qi et al. (2021) [24], concentrations of ruminal free amino acids were determined. Rumen fluid (2 mL) was centrifuged (13,500× *g*, 4 °C, 15 min). Acetonitrile (300 μL) was added to the supernatant, subsequently vortexed for 15 s, and then centrifuged (13,500× *g*, 4 °C, 5 min). Ultimate ultrafiltrates were analyzed using an automated amino acid analysis system (L-8900, Hitachi High-Technologies Co., Tokyo, Japan). Qualitative and quantitative determination of individual free amino acids was achieved through comparative analysis of chromatographic peak areas relative to the reference signal generated by an external calibrant.

### 2.8. Rumen Microorganisms

Rumen fluid samples stored at −80 °C were sent to Majorbio Bio-Pharm Technology Co., Ltd. (Shanghai, China) for microbiome analysis. Total microbial genomic DNA was extracted using the Bacterial Genomic DNA Extraction Kit (Omega Bio-tek, Norcross, GA, USA). The quality and concentration of DNA were determined by 1.0% agarose gel electrophoresis and a NanoDrop2000 spectrophotometer (Thermo Scientific, Wilmington, DE, USA) and kept at −80 °C prior to further use. The V3-V4 hypervariable region of the bacterial 16S rRNA gene was amplified with primers 338F (5′-ACTCCTACGGGAGGCAGCAG-3′) and 806R (5′-GGACTACHVGGGTWTCTAAT-3′) using a T100 Thermal Cycler PCR thermocycler (BIO-RAD, Hercules, CA, USA). PCR amplification cycling conditions were as follows: initial denaturation at 95 °C for 3 min, followed by 27 cycles of denaturing at 95 °C for 30 s, annealing at 55 °C for 30 s, and extension at 72 °C for 45 s, as well as single extension at 72 °C for 10 min, ending at 4 °C. The PCR product was extracted from 2% agarose gel and purified using a PCR Clean-Up Kit (YuHua, Shanghai, China) according to the manufacturer’s instructions and quantified using Qubit 4.0 (Thermo Fisher Scientific, USA). The amplified DNA fragments underwent purification followed by paired-end sequencing on the Illumina MiSeq PE250 platform (Illumina, San Diego, CA, USA). Quality filtering, clustering, and analysis of the sequencing data were processed using the Novogene Magic platform (Novogene, Beijing, China). High-throughput sequencing was then performed to analyze the rumen microbial composition and function. Raw sequences were submitted to the NCBI Sequence Read Archive (SRA) (accession number PRJNA1307509). 

### 2.9. Statistical Analysis

Microsoft Excel 2015 was used to collect and organize the fattening sheep’s growth performance, apparent nutrient digestibility, and rumen fermentation parameter data. Data on ruminal pH were analyzed by first converting pH values to hydrogen ion concentration ([H^+^] = 10^(−pH)^). Statistical analyses were performed on the [H^+^] values, and the resulting means were reconverted to the pH scale for presentation in tables and figures. All other data were analyzed directly. IBM SPSS Statistics v22.0 software was used to statistically analyze and test the significance of differences from each treatment group via one-way analysis of variance (ANOVA). The statistical model was X_i__j_ = μ + α_i_ + ε_i__j_ (X_i__j_ is the observed value, μ is the overall mean, α_i_ is the fixed effect of the *i*th dietary treatment, and ε_ij_ is the random error term). Duncan’s multiple range test was used for multiple comparisons. Orthogonal polynomial contrasts were used to test for linear and quadratic trends across the RP-Leu supplementation levels. A probability value of *p* < 0.05 (two-tailed) was considered statistically significant. Figures were plotted using GraphPad Prism software (version 10.1.2). Analysis of microbial sequencing data was conducted on the MagicBi Platform (Majorbio, Shanghai, China). Based on the operational taxonomic units (OTUs) information, rarefaction curves and alpha diversity indices were calculated with Mothur v1.30.1. The similarity among the microbial communities in different samples was determined using principal coordinate analysis (PCoA) based on Bray–Curtis dissimilarity. The linear discriminant analysis (LDA) effect size (LEfSe) was performed to identify the significantly abundant taxa (phylum to genera) of bacteria among the different groups. The analysis of the potential relationships between microbial genera and fermentation metabolites was performed on the MagicBi Platform (Majorbio, Shanghai, China) using the built-in statistics module. The correlation matrix was plotted as a heatmap, with significance levels set at *p* < 0.05 and *p* < 0.01.

## 3. Results

### 3.1. Growth Performance

Supplementation with RP-Leu improved the growth performance of fattening sheep (Table 2). Orthogonal polynomial contrast analysis revealed a significant linear increase in average daily gain (ADG) with increasing RP-Leu supplementation (*p* < 0.01 for linear contrast). Sheep supplemented with 1.0 g/d RP-Leu achieved the highest growth rate, with an ADG of 193.73 g/d, which was 22.2% greater than that of the L-0 group (158.53 g/d; *p* < 0.05). The feed-to-gain ratio (F:G ratio) was significantly reduced in the L-1.0 and L-1.5 groups versus the L-0 group (*p* < 0.05), and the lowest F:G ratio (4.59) was seen in the L-1.0 group compared to the L-0 group (5.74). The F:G ratio also exhibited a significant quadratic trend (*p* < 0.05). Furthermore, a significant linear increase in final body weight was observed with RP-Leu supplementation (*p* < 0.05), while RP-Leu supplementation had no statistically significant alteration in ADMI (*p* > 0.05). This indicated that improved feed conversion efficiency was correlated with RP-Leu supplementation.

### 3.2. Apparent Nutrient Digestibility

Supplementation with RP-Leu had no effect on apparent nutrient digestibility in the whole digestive tract of fattening sheep (Table 3). As shown in the table, dry matter (DM), neutral detergent fiber (NDF), acid detergent fiber (ADF), crude protein (CP), and ether extract (EE) digestibility remained unaffected by dietary treatments (*p* > 0.05). Furthermore, orthogonal polynomial contrast analysis revealed no significant linear or quadratic trends (*p* > 0.05) for any of the measured digestibility parameters across the supplementation levels.

### 3.3. Rumen Fermentation Parameters

Supplementation with RP-Leu altered ruminal fermentation parameters in fattening sheep (Table 4). Propionate concentration increased significantly in the L-1.5 group (*p* < 0.05) and increased by 15.61% compared to that of the L-0 group (26.15 mmol/L vs. 22.62 mmol/L). Notably, an orthogonal contrast analysis revealed a significant linear and quadratic increase (*p* < 0.01) in propionate concentration with increasing RP-Leu supplementation. Meanwhile, isovalerate concentrations were dramatically elevated across all supplementation groups, showing an increase of approximately 50–60% compared to the L-0 group (*p* < 0.01), which also exhibited a strong dose-dependent response, as evidenced by significant linear and quadratic contrasts (*p* < 0.01). The acetate-to-propionate ratio (A:*p* ratio) exhibited a statistically significant reduction in the L-1.5 group (*p* < 0.05), with a contrast analysis confirming a significant linear decrease (*p* = 0.01) across treatment groups, while the ratio in the L-0.5 and L-1.0 groups showed a tendency to be lower but not significantly different from that of the L-0 group (*p* > 0.05). Although NH_3_-N, acetic acid, butyric acid, valeric acid, and TVFA concentrations increased among RP-Leu-supplemented groups, no statistical significance was observed across treatment groups (*p* > 0.05). However, a contrast analysis indicated a significant linear decrease in acetic acid (*p* = 0.04) and a linear increase in valeric acid (*p* = 0.02) with increasing RP-Leu supplementation.

### 3.4. Free Amino Acid Concentrations in Rumen Fluid

**Figure 1:**
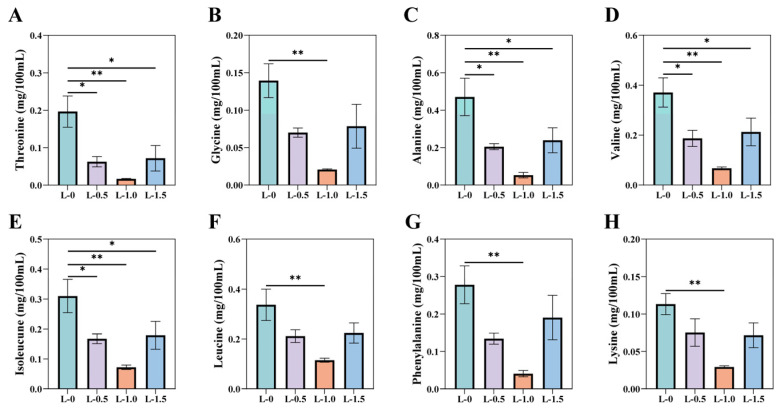
Effects of RP-Leu on ruminal amino acid composition (n = 4). (**A**) Threonine; (**B**) leucine; (**C**) valine; (**D**) alanine; (**E**) isoleucine; (**F**) phenylalanine; (**G**) lysine; (**H**) glycine. Data are expressed as mean ± SEM. Significance is denoted by asterisks: * *p* < 0.05, ** *p* < 0.01.

As shown in Figure 1, RP-Leu altered the concentration of free amino acids in the rumen of fattening sheep. Compared to the L-0 group, all RP-Leu-supplemented groups exhibited reduced concentrations of BCAA in rumen fluid (*p* < 0.05), including leucine, valine, and isoleucine, and maximal reductions were observed in the L-1.0 group (*p* < 0.01, Figure 1B,C,E). RP-Leu supplementation also significantly reduced the concentrations of a variety of other amino acids measured (*p* < 0.05). Threonine, alanine, phenylalanine, lysine, and glycine concentrations were consistently lower in the RP-Leu groups versus the L-0 group (*p* < 0.05), most markedly in the L-1.0 group (*p* < 0.01; Figure 1A,D,F–H).

### 3.5. Rumen Microbiota

**Figure 2:**
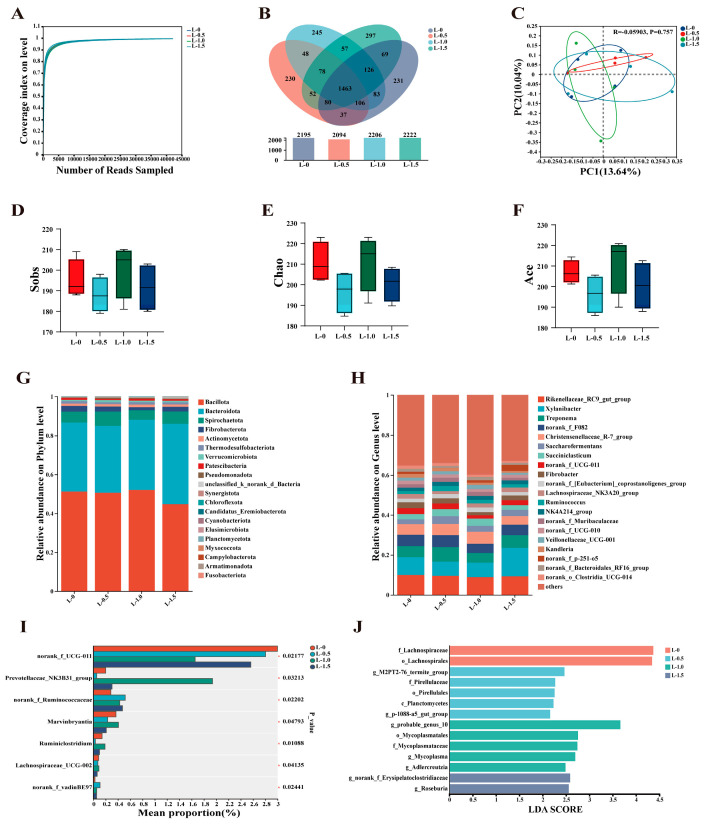
Effects of RP-Leu on rumen microbial community composition (n = 4). (**A**) Rarefaction curves indicating sequencing depth adequacy. (**B**) Venn diagram depicting the distribution of exclusive and common operational taxonomic units (OTUs) across experimental cohorts. (**C**) Principal coordinates analysis (PCoA) plot visualizing β-diversity and inter-group dissimilarity. (**D**–**F**) α-Diversity indices (Sobs, Chao, and Ace) evaluating microbial richness and evenness. (**G**) Phylum-level community composition. (**H**) Genus-level community composition. (**I**) Differential abundance analysis showing significant differences in taxon abundance between groups. (**J**) Linear discriminant analysis (LDA) coupled with effect size (LEfSe) identifying differentially abundant microbial taxa across groups. Significance is indicated by asterisks: * *p* < 0.05.

16S rRNA gene sequencing revealed that supplementation with RP-Leu optimized the structure of the rumen microbial community (Figure 2). Rarefaction curves tended to flatten at the end, indicating that the sequencing depth was sufficient and the data were reasonable (Figure 2A). A Venn analysis showed that there were 237, 230, 246, and 299 unique species at the OTU level in the L-0, L-0.5, L-1.0, and L-1.5 groups, respectively (Figure 2B). The Sobs, Chao, and Ace indices (reflecting the microbial richness) were not significantly different among the treatment groups (*p* > 0.05, Figure 2D–F), indicating that RP-Leu supplementation did not alter the richness of rumen microbial communities. PcoA analysis further confirmed that there were no discernible differences in microbial community structure (Beta diversity) between groups (*p* > 0.05, Figure 2C). *Bacillota* and *Bacteroidota* were the two dominants at the phylum level, accounting for more than 84% of the total 16S rRNA gene sequences (Figure 2G). At the genus level, *Rikenellaceae_RC9_gut_group*, *Xylanibacter*, and *Treponema* were the predominant genera (Figure 2H). A one-way ANOVA analysis of rumen microbial species composition showed that the relative abundance of *norank_f_UCG-011* was significantly lower in the RP-Leu-added groups versus the L-0 group (*p* < 0.05), and the relative abundance of *norank_f_Ruminococcaceae* and *Prevotellaceae_NK3B31_group* increased significantly (*p* < 0.05, Figure 2I). Core microbial taxa underwent the linear discriminant analysis LEfSe profiling, revealing the following treatment-specific enrichments: *g_M2PT2-76_termite_group* was enriched specifically in the L-0.5 group, *g_probable_genus_10* was enriched specifically in the L-1.0 group, and L-1.5 group-specific enrichment bacteria were *g_norank_f_Erysipelatostridiaceae* and *g_Roseburia* (Figure 2J).

### 3.6. Correlations Between Rumen Fermentation Parameters, Free Amino Acids, and Microbiota

**Figure 3:**
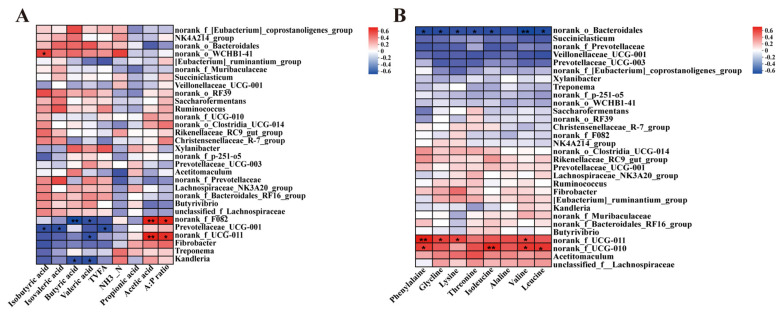
Genus-level covariation patterns between ruminal microbiota and fermentation parameters and free amino acids quantified via Spearman’s correlation analysis. (**A**) Heatmap of Spearman correlations between rumen microbiota (genus level) and rumen fermentation parameters. NH_3_-N: ammonia nitrogen; A:P ratio: acetate-to-propionate ratio; TVFA: total volatile fatty acids. (**B**) Heatmap of Spearman correlations between rumen microbiota (genus level) and ruminal free amino acids. Significance is indicated by asterisks: * *p* < 0.05, ** *p* < 0.01.

Spearman’s correlation analysis revealed significant associations between rumen microbial communities (genus level) and key fermentation parameters and free amino acid concentrations (Figure 3). Microbial genera demonstrated significant covariation patterns with fermentation metabolites: acetic acid exhibited strong positive associations with *norank_f_F082* and *norank_f_UCG-011* (*p* < 0.01, Figure 3A). Conversely, isovaleric and isobutyric acids manifested inverse relationships with *Prevotellaceae_UCG-001* (*p* < 0.05). The A:P ratio displayed positive correlations with *norank_f_F082* and *norank_f_UCG-011* (*p* < 0.05). A Spearman analysis of ruminal free amino acids (Figure 3B) identified seven amino acids showing negative correlations with *norank_o_Bacteroidales* (*p* < 0.05), including phenylalanine, glycine, lysine, threonine, isoleucine, valine, and leucine. Among these, phenylalanine, glycine, lysine, and valine demonstrated positive linkages with *norank_f_UCG-011* (*p* < 0.05). Phenylalanine, isoleucine, valine, and leucine covaried positively with *norank_f_UCG-010* (*p* < 0.05).

## 4. Discussion

### 4.1. Effects of RP-Leu on Growth Performance and Nutrient Apparent Digestibility

Leu cannot be synthesized by the animals or the amount synthesized is not enough to meet the growth demand, so it must be supplied via dietary intake, as it plays an indispensable role in the process of animal growth and development [25]. Studies have shown that the supplementation of 17.45 g/d RP-Leu to the diets of dairy goats increased ADG by approximately 15%, decreased feed intake, and improved the F:G ratio [26]. Similarly, adding 1.435 g/L Leu to the diet of preweaning calves increased their ADG by approximately 0.38% [27]. In this experiment, the supplementation of RP-Leu increased ADG and decreased the feed conversion ratio in fattening sheep. Additionally, a contrast analysis revealed both a significant linear increase and a significant quadratic effect in the F:G ratio with increasing RP-Leu supplementation. This indicates that while the F:G ratio generally improved with dose, the rate of improvement slowed at higher levels. Supplementation with RP-Leu enhanced feed utilization efficiency rather than increased intake, as shown by the elevated ADG but unchanged ADMI in growth performance. In addition, there was no significant difference in the apparent digestibility of nutrients, which further indicates that the promotion of growth by RP-Leu is not achieved by enhancing the digestion of macro-nutrients, but it may be realized by optimizing rumen fermentation patterns, improving host metabolic efficiency and energy allocation. This mechanism is supported by a number of studies [28,29,30].

### 4.2. Effects of RP-Leu on Rumen Fermentation Patterns

The rumen of ruminants can degrade dietary nutrients and produce acetic acid and propionic acid [31]. Propionic acid is the substance in the organism that can produce glucose, and the conversion of its carbon skeleton to glucose is more efficient than that of acetic acid; when propionic acid levels are elevated, the organism is able to utilize more effective energy, thus promoting animal growth [32]. The rumen fermentation pattern is often marked by the A:P ratio, and when the A:P ratio decreases, it tends toward a propionic acid fermentation pattern, while when the A:P ratio increases, it tends toward an acetic acid fermentation pattern [33]. Our results demonstrate that dietary RP-Leu at 1.5 g/d elevated propionic acid and isovaleric acid concentrations by 15.6% and 51.1%, respectively, while concurrently reducing the A:P ratio by 25.7% (from 4.47 to 3.32) compared to the control, indicating that the rumen fermentation pattern was changed and tended to favor the propionic acid fermentation pattern. These observations align with empirical evidence reported in prior investigations [10,11,34]. In addition, an integrated analysis of growth performance and ruminal fermentation parameters revealed optimal outcomes at the 1.0 g/d RP-Leu supplementation level, suggesting this dosage to be the potential optimum for fattening sheep.

### 4.3. Effects of RP-Leu on Ruminal Free Amino Acid

Ruminal free amino acids are mainly derived from the degradation of dietary proteins [35], which are both precursors of MCP [36] and can be used as a readily available energy source for microorganisms when there is a lack of fermentable carbohydrates [37]. Ruminal free amino acid catabolism proceeds primarily through two distinct biochemical pathways: oxidative deamination, wherein specific amino acids function as electron donors, and reductive deamination, where complementary amino acids serve as electron acceptors, ultimately yielding NH_3_-N and short-chain VFAs [38]. RP-Leu reduced the content of BCAAs in the rumen. This effect likely stems from the delayed release of leucine by RP-Leu in the rumen, creating a state of “leucine pseudo-deficiency” and a decrease in rumen Leu and other amino acids. Following delayed release, a portion of RP-Leu further stimulated the microbial deamination of BCAAs within the rumen, leading to their conversion into branched-chain volatile fatty acids (BCVFAs) in vivo. This process resulted in elevated ruminal concentrations of butyrate and isovalerate. These findings indicate that RP-Leu reconfigured ruminal amino acid metabolism. However, the supplementation of RP-Leu in fattening cattle diets resulted in elevated ruminal phenylalanine and lysine while decreasing cysteine [12], and the difference that caused this result may be species-specific.

### 4.4. Effects of RP-Leu on the Rumen Microbiota

Variations in rumen fermentation parameters and patterns exhibit significant associations with rumen microbial composition and structure [39,40]. The rumen microbiota is predominantly composed of *Bacillota* and *Bacteroidota* [41]. Among them, *Bacillota* plays an important role in energy utilization [42,43], while *Bacteroidota* is associated with the degradation of proteins and carbohydrates and the synthesis of propionic acid in the rumen [44,45]. Dietary RP-Leu administration in this present investigation elicited no statistically significant alterations in the relative abundances of *Bacillota* or *Bacteroidota* at the phylum rank, indicating that the supplementation of RP-Leu had no effect on rumen microbial diversity in fattening sheep. This finding corroborated empirical outcomes documented in the extant literature [10,12]. *norank_f_UCG-011* carries cellulase and hemicellulase genes [46], which are mainly closely related to rumen acetate production and positively correlated with acetate concentration [47]. *Prevotellaceae_NK3B31_group* is classified under the family *Prevotellaceae*, which is one of the predominant categories of fiber-degrading bacteria in ruminant rumen. It efficiently decomposes hemicellulose, pectin, and other plant structural polysaccharides [48], and its elevated abundance is usually associated with an animal’s ability to efficiently utilize the diet [49]. In this study, the L-1.0 group exhibited a marked reduction in the relative abundance of *norank_f_UCG-011* alongside an increased abundance of *Prevotellaceae_NK3B31_group* versus the L-0 group. And based on the LEfSe analysis, *g_probable_genus_10* was specifically enriched in the L-1.0 group. Notably, in vitro rumen fermentation experiments confirmed that *g_probable_genus_10* could degrade fiber and produce VFA [12], showing that RP-Leu promoted the proliferation of efficient fiber-degrading bacteria in the rumen. This finding is mutually corroborative with the elevated ruminal isovalerate concentration observed in the L-1.0 group, as isovalerate serves as a critical growth factor for fiber-degrading bacteria [12,14]. Therefore, we speculate that the small amount of leucine released from RP-Leu in the rumen was converted to isovalerate, which subsequently stimulated the enrichment of specific fiber-degrading bacteria. The Spearman correlation analysis showed that acetic acid and the A:P ratio were positively correlated with *norank_f_F082* and *norank_f_UCG-011*. Empirical investigations have documented positive associations between norank_f_F082 abundance and propionate concentrations, concomitant with inverse correlations with methanogenesis [50,51]. Supplementation with Chinese herbal residues in goat diets demonstrated negative correlations between *norank_f_F082* abundance and growth performance [52], and this discrepancy relative to the present findings may be attributable to distinct dietary supplement formulations employed. In this experiment, *norank_o_Bacteroidales* was negatively correlated with the rumen free amino acids phenylalanine, glycine, lysine, threonine, isoleucine, valine, and leucine, and *norank_f_UCG-011* was positively correlated with phenylalanine. Other studies found that *Bacteroidales_UCG_001*, a taxon related to *norank_o_Bacteroidales* and *norank_f_UCG-011*, was synchronized with the enrichment of valine, leucine, and isoleucine degradation pathways [53], suggesting that *norank_o_Bacteroidales* and *norank_f_UCG-011* can regulate rumen amino acids. This suggests that rumen microbial changes are associated with shifts in rumen fermentation parameters and fermentation patterns.

## 5. Conclusions

RP-Leu supplementation enhanced growth performance in fattening sheep by modulating rumen fermentation parameters and shifting fermentation patterns toward propionate dominance, and the optimal supplementation level was determined to be 1.0 g/d. Furthermore, RP-Leu supplementation reduced the ruminal concentrations of free branched-chain amino acids and enriched the abundance of fiber-degrading bacteria. This study’s findings provide new insights into the effects of RP-Leu on the growth performance and rumen fermentation function of fattening sheep, as well as serving as a valuable reference for the application of leucine.

## Figures and Tables

**Table 1:** Basic diet composition and nutritional components (dry matter basis).

Items	Content	Nutritional Composition, %			Content
Ingredients, %		Crude protein			11.97
Corn straw	17.00	Crude fat			1.90
Alfalfa hay	33.00	Crude ash			8.76
Corn	25.06	Neutral detergent fiber			57.57
Wheat	3.26	Acid detergent fiber			24.20
Soybean meal 43%	3.26	Calcium			0.70
Cottonseed meal 50%	3.97	Phosphorus			0.28
Corn germ meal	3.80	Leucine			0.78
Corn bran	7.30	Metabolizable Energy (MJ/kg)			9.64
Limestone powder	0.70				
Soybean oil	0.38				
NH_4_Cl	0.27				
NaCl	0.50				
NaHCO_3_	0.50				
Premix	1.00				
Total	100.00				

The metabolic energy was calculated according to the formula in the table of “Nutritional Requirements of Meat Sheep” (NY/T 816-2021): ME (MJ/kg DM) = 0.0460.820 × DE (MJ/kg DM); DE(MJ/kg DM) = 17.2110.135 × NDF(%DM), as well as the other indices are measured values. The premix provided the following per kilogram of complete diet: 10,000 IU of vitamin A, 1000 IU of vitamin D, 200 IU of vitamin E, 145 mg of iron, 80 mg of zinc, 20 mg of copper, 98 mg of manganese, 2.5 mg of iodine, 0.35 mg of selenium, and 0.65 mg of cobalt.

**Table 2:** Effects of RP-Leu on growth performance in fattening sheep.

Items	Groups				SEM	*p*-Value	Contrast *p*-Value	
	L-0	L-0.5	L-1.0	L-1.5			Linear	Quadratic
IBW, kg	18.74	19.23	18.96	19.44	0.38	0.93	0.61	0.99
FBW, kg	28.25	29.34	30.58	30.67	0.46	0.2	0.04	0.58
ADMI, kg/d	0.91	0.87	0.89	0.93	0.03	0.88	0.67	0.66
ADG, g/d	158.53 ^c^	168.55 ^bc^	193.73 ^a^	187.10 ^ab^	4.44	<0.01	<0.01	0.30
F:G ratio	5.74 ^a^	5.16 ^ab^	4.59 ^b^	4.97 ^b^	0.15	<0.01	<0.01	0.04

IBW: initial body weight; FBW: final body weight; ADMI: average dry matter intake; ADG: average daily weight gain; F:G ratio: feed-to-gain ratio; SEM: standard error of the mean. ^a,b,c^ Different superscript letters within the same row indicate significant differences (*p* < 0.05). *p*-values from orthogonal polynomial contrast analysis are presented for linear and quadratic trends.

**Table 3:** Effects of RP-Leu on apparent nutrient digestibility in fattening sheep.

Items	Groups				SEM	*p*-Value	Contrast *p*-Value	
	L-0	L-0.5	L-1.0	L-1.5			Linear	Quadratic
DM, %	68.15	70.76	71.2	72.37	0.98	0.58	0.23	0.75
NDF, %	57.48	58.6	56.92	59.27	0.67	0.71	0.62	0.71
ADF, %	44.4	36.55	45.26	43	2.03	0.51	0.82	0.54
CP, %	71.38	74.06	66.05	72.6	2.21	0.22	0.41	0.22
EE, %	77.09	80.53	80.89	84.91	2.15	0.75	0.34	0.96

DM: dry matter; NDF: neutral detergent fiber; ADF: acid detergent fiber; CP: crude protein; EE: ether extract; SEM: standard error of the mean. *p*-values from orthogonal polynomial contrast analysis are presented for linear and quadratic trends.

**Table 4:** Effects of RP-Leu on rumen fermentation parameters in fattening sheep.

Items	Groups				SEM	*p*-Value	Contrast *p*-Value	
	L-0	L-0.5	L-1.0	L-1.5			Linear	Quadratic
pH	7.49	7.51	7.45	7.41	0.04	0.86	0.47	0.75
NH_3_-N, mg/L	100.11	131.00	113.16	123.53	4.80	0.1	0.18	0.24
Acetic acid, mmol/L	107.24	107.33	86.19	86.48	4.52	0.13	0.04	0.99
Propionic acid, mmol/L	22.62 ^b^	21.94 ^b^	21.90 ^b^	26.15 ^a^	0.6	<0.01	<0.01	<0.01
Isobutyric acid, mmol/L	1.29	1.45	1.49	1.45	0.07	0.81	0.47	0.55
Butyric acid, mmol/L	12.19	12.22	12.96	13.23	0.39	0.78	0.35	0.9
Isovaleric acid, mmol/L	1.41 ^b^	2.09 ^a^	2.30 ^a^	2.13 ^a^	0.11	<0.01	<0.01	<0.01
Valeric acid, mmol/L	0.58	0.64	0.69	0.74	0.03	0.12	0.02	0.98
A:P ratio	4.47 ^a^	4.92 ^a^	3.94 ^ab^	3.32 ^b^	0.25	0.04	0.01	0.30
TVFA, mmol/L	145.32	145.67	125.53	130.18	4.09	0.18	0.08	0.77

NH_3_-N: ammonia nitrogen; A:P ratio: acetate-to-propionate ratio; TVFA: total volatile fatty acids; SEM: standard error of the mean. ^a,b^ Different superscript letters within the same row indicate significant differences (*p* < 0.05). *p*-values from orthogonal polynomial contrast analysis are presented for linear and quadratic trends.

## Data Availability

The original contributions presented in this study are included in the article. Further inquiries can be directed at the corresponding author.
